# Posterior reversible encephalopathy syndrome in a bilateral lung transplant recipient with subtherapeutic tacrolimus levels: a case report

**DOI:** 10.1186/s13256-026-05964-6

**Published:** 2026-03-22

**Authors:** Aashish Maurya, Arthas Flabouris

**Affiliations:** 1 General Medicine Unit, Royal Adelaide Hospital, Adelaide, South Australia Australia; 2 Intensive Care Unit, Royal Adelaide Hospital, Adelaide, South Australia Australia; 3 Faculty of Health and Medical Science, Adelaide Medical School, The University of Adelaide, Adelaide, South Australia Australia

**Keywords:** Lung transplant, Posterior reversible encephalopathy syndrome, Tacrolimus, Seizure, Blindness

## Abstract

**Background:**

Posterior reversible encephalopathy syndrome is a rare neurological disorder typically associated with hypertension, cytotoxic agents, or supratherapeutic immunosuppressant levels. We report what appears to be the first documented case of posterior reversible encephalopathy syndrome in a bilateral lung transplant recipient occurring with subtherapeutic tacrolimus levels.

**Case presentation:**

A 59-year-old white male with prior bilateral lung transplantation and multiple risk factors, including sepsis, corticosteroid use, and acute kidney injury, presented with sudden bilateral cortical blindness and seizures, initially concerning for stroke. Tacrolimus levels were subtherapeutic. Rapid diagnosis of posterior reversible encephalopathy syndrome was made using computed tomography stroke perfusion imaging. Intensive blood pressure control and substitution of tacrolimus with cyclosporin led to complete neurological recovery.

**Conclusion:**

This case highlights the multifactorial nature of posterior reversible encephalopathy syndrome and the need for vigilance even when immunosuppressant levels are subtherapeutic. Early recognition and timely management can prevent irreversible neurological injury, with important implications for transplant care and immunosuppressive therapy monitoring.

## Introduction

Posterior reversible encephalopathy syndrome (PRES), first described by Hinchey *et al*., is a clinico-radiological diagnosis characterized by typical clinical features, associated risk factors, and distinctive brain computed tomography (CT) and magnetic resonance imaging (MRI) findings [1–3]. Onset can be acute or subacute, with symptoms developing over a period of hours to weeks [4].

PRES presents with a range of neurological symptoms, including visual loss (blurred vision or cortical blindness), headache, altered consciousness, seizures, and focal neurological deficits (for example, weakness, sensory loss, or speech disturbance) [1, 2, 5]. Tacrolimus, an immunosuppressive medication, is highly effective in preventing organ rejection and prolonging graft survival [1]. The development of PRES can occur at sub-, normal, and supratherapeutic tacrolimus levels [6–9]. Association with supratherapeutic tacrolimus levels is well described in literature, with incidence following solid organ transplantation ranging between 0.5% and 5%, and approximately 2% after lung transplantation [2, 5, 8–11]. Herein, we report a case of PRES in a bilateral lung transplant recipient precipitated by subtherapeutic tacrolimus levels.

## Case report

A 59-year-old white male presented to a trauma center following a high-speed motor vehicle accident. He sustained multiple injuries, including a traumatic brain injury requiring surgical intervention under orthopedic and plastic surgery teams. His past medical history was significant for bilateral sequential lung transplantation for severe chronic obstructive pulmonary disease, performed 3 years prior to admission. He had no history of seizure disorder, hypertension, or drug or alcohol misuse. His regular medications included tacrolimus 3 mg twice daily, azathioprine 100 mg daily, and prednisolone 12.5 mg daily for immunosuppression.

**Fig. 1:**
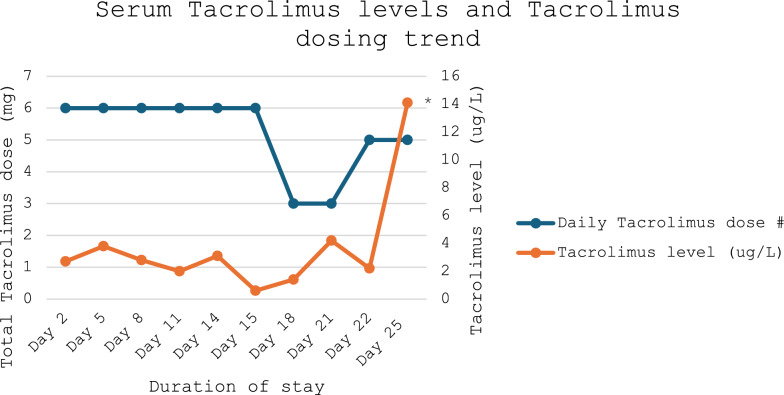
Serum tacrolimus levels and tacrolimus dosing trend from admission to code stroke event/posterior reversible encephalopathy syndrome development (day 25). Therapeutic tacrolimus levels range between 5 ug/L and 15 ug/L. ^#^Daily tacrolimus dose does not indicate the frequency at which tacrolimus was administered (for example, twice/once a day). ^*^Tacrolimus level 14.7 ug/L is falsely elevated as it was taken 3 hours after dose administration

He was admitted directly to the intensive care unit (ICU) and underwent a total of seven surgeries over 3 weeks. During his admission, he developed steroid-induced hyperglycemia, acute kidney injury, and a deep tissue infection, which were managed with piperacillin/tazobactam, azithromycin, and vancomycin. Tacrolimus dosing varied throughout the admission, and serum tacrolimus levels were predominantly subtherapeutic (below 5–15 µg/L) (Fig. 1).

**Fig. 2:**
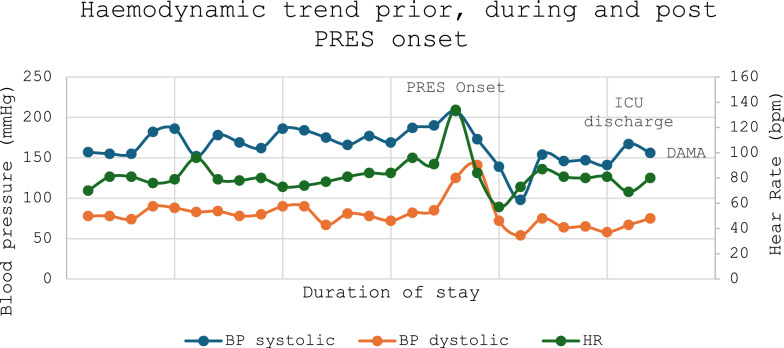
Hemodynamic stability in the days leading up to posterior reversible encephalopathy syndrome development compared with during onset, intensive care unit discharge, and discharge against medical advice

**Fig. 3:**
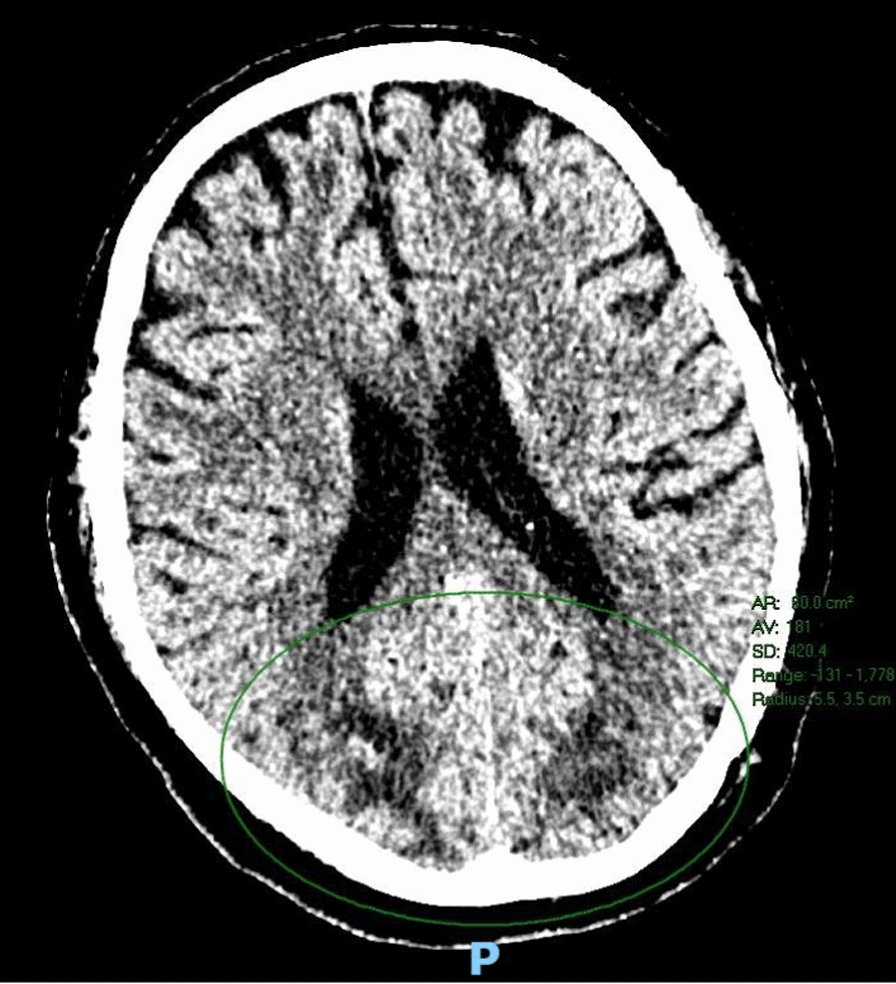
Computed tomography of brain perfusion (code-stroke) demonstrating abnormal predominantly subcortical hypoattenuation in bilateral occipital lobes (highlighted with a green circle) with new reduced blood mean transit time and increased cerebral blood volume. The radiological report did not quantify the mean transit time or cerebral blood volume

**Fig. 4:**
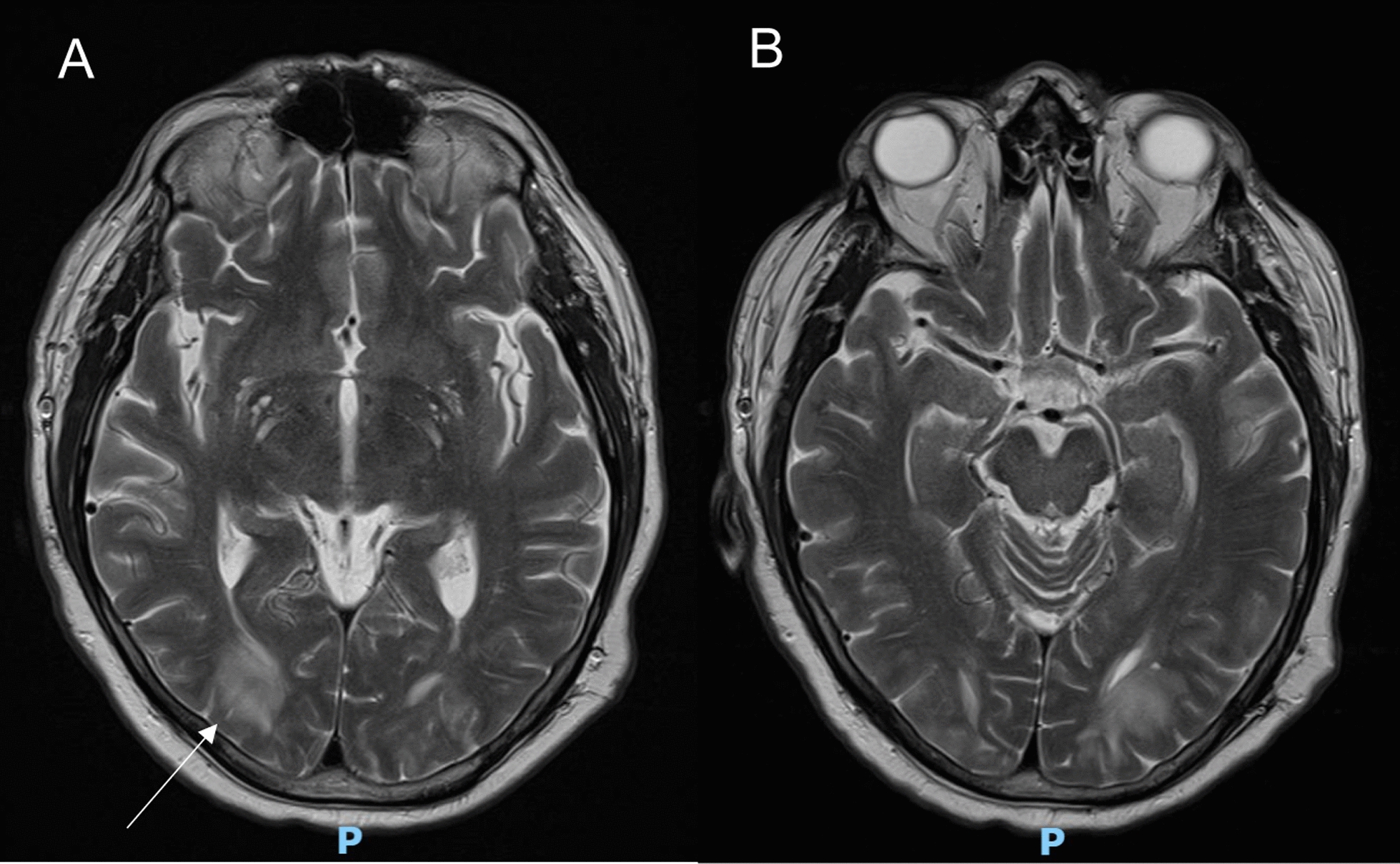
Coronal T2-weighted fluid-attenuated inversion recovery magnetic resonance imaging of the brain showing hyperintensity and mild volume expansion within deep and subcortical white matter of both posterior occipital lobes. **A** Hyperintense lesion in right occipital (white arrow). **B** Hyperintense lesion in left occipital (white arrow)

On day 25 of his hospital stay, progressive hypertension was noted (Fig. 2) hours prior to the sudden onset of bilateral cortical visual loss. This raised concerns for an acute stroke, prompting activation of the rapid response team and stroke team. On initial review, the National Institutes of Health (NIH) Stroke Scale score was 0, and the blood pressure was 187/82 mmHg. A stroke-series computed tomography (CT) of the brain and perfusion study demonstrated subcortical hypoattenuation in the bilateral occipital lobes, suggestive of PRES (Fig. 3), which was confirmed on follow-up MRI of the brain (Fig. 4).

Shortly thereafter, the patient developed encephalopathy, tonic–clonic seizures, worsening hypertension (208/125 mmHg), and tachycardia (134 beats per minute), requiring ICU admission for seizure monitoring, encephalopathy management, and blood pressure control. Postictal blood tests demonstrated worsening renal function (estimated glomerular filtration rate [eGFR] 34 mL/minute/1.73 m^2^ and serum creatinine 185 µmol/L), lactic acidosis (14.8 mmol/L), hypokalemia (2.9 mmol/L), and normal magnesium (0.79 mmol/L).

Initial blood pressure and tachycardia were managed with intravenous metoprolol, and the patient was loaded with intravenous levetiracetam prior to ICU transfer. During ICU stay, oral amlodipine 10 mg daily, perindopril 10 mg daily, and moxonidine 200 µg twice daily were administered to control blood pressure. The patient experienced no further seizures in the ICU, and regular antiepileptics were not commenced.

Corticosteroids were continued, while tacrolimus was switched to cyclosporin with consultation with the local lung transplant specialists, with the dose titrated up to 150 mg twice daily. The patient made a complete neurological recovery with no focal deficits on serial examinations after cessation of tacrolimus. He spent 5 days in ICU but remained hypertensive (systolic blood pressure [BP] > 140 mmHg). He ultimately self-discharged against medical advice 17 days after ICU admission (Fig. 2). On serial outpatient follow-up, no long-term neurological deficits were identified. However, there was a significant deterioration in lung function following an influenza A infection while on cyclosporine. Tacrolimus was not re-challenged, and the patient remains on cyclosporine.

## Discussion

Posterior reversible encephalopathy syndrome (PRES) is a rare but serious neurotoxic complication in transplant recipients, commonly reported following kidney, liver, and bone marrow transplantation. It presents with variable neurological symptoms, including visual disturbance, seizures, focal neurological deficits, and altered mental status. It typically involves the middle cerebral artery–posterior cerebral artery border zone of the occipital and parietal lobes [12]. Neuroimaging is essential for the diagnosis, with typical findings of bilateral cortical–subcortical edema of posterior cerebral white matter [2–5, 13–15]. Abnormalities in parieto-occipital, frontal, temporal, thalamic, cerebellar, brainstem, and basal ganglia regions have also been reported [16].

The pathophysiology of PRES is poorly understood. However, there are two common hypotheses [1, 4, 5, 9].

1. Disordered cerebrovascular autoregulation causing endothelial injury from abrupt blood pressure fluctuations and resultant vasogenic edema. Notably, approximately 20% of patients with PRES are not hypertensive, and mean arterial pressure rarely exceeds the upper autoregulatory limit [16].
2. Vasoconstriction and hypoperfusion, leading to ischemic cytotoxic injury and vasogenic edema.

Histopathology of brain tissue has demonstrated increased expression of vascular endothelial growth factor inferring with increased permeability of the blood–brain barrier [16]. Vasogenic edema manifests as subcortical and cortical edema on brain imaging [1, 2]. Although PRES is typically reversible, it carries a mortality of 19% and a morbidity of 44% when not managed promptly [2]. A minority of cases demonstrate persistent or recurrent neurological injury [16].

PRES in solid organ transplantation with tacrolimus can be multifactorial, and previous case reports have described additional contributing factors [6, 7, 9, 14]. Common risk factors include uncontrolled hypertension, renal or hepatic failure, eclampsia, solid organ transplantation, immunosuppression, sepsis, and cytotoxic medications [1, 2]. In this patient, risk factors included hypertension, sepsis, solid organ transplantation, acute kidney injury, corticosteroid use, and tacrolimus exposure. Tacrolimus is among the most common drugs associated with PRES [10]. While supratherapeutic levels are classically implicated, PRES can occur with subtherapeutic levels, as seen in this case [5, 9, 10]. This variability is thought to result from polymorphisms affecting drug metabolism. Normally, tacrolimus does not cross the blood–brain barrier because the P-glycoprotein efflux pump prevents its entry. In some patients, reduced or absent P-glycoprotein activity allows tacrolimus penetration, causing neurotoxicity [5, 9]. Brain biopsies have confirmed tacrolimus-induced demyelination of nerve axons [14, 15].

There are no formal guidelines or randomized control trials on management of PRES, regardless of the trigger [2, 5]. Recommended management includes prompt blood pressure control, electrolyte replacement (especially magnesium), seizure management and prophylaxis, and withdrawal or dose reduction of the offending agent [5]. Up to 70% of patients with PRES, particularly those with severe hypertension, depressed level of consciousness, or seizures, are admitted to an intensive care unit [17]. Similar to this case, a common measure in published cases is switching tacrolimus to cyclosporine, in cases of intolerance or adverse effects to other calcineurin inhibitors [7–9]. Both these agents are of similar class of drugs and suggested to share similar mechanism of action.

Several case reports have described PRES secondary to hypertension, trauma, or tacrolimus in transplant recipients (kidney, liver, stem cell, and bone marrow). However, to our knowledge, this is the first reported case of PRES in a bilateral lung transplant recipient precipitated by subtherapeutic tacrolimus levels. A strong correlation was observed with resolution in symptoms on ceasing of tacrolimus, which has been universally described in other case reports of tacrolimus-associated PRES in solid organ transplant receipts [7, 9].

PRES can mimic acute stroke. In this case, rapid activation of the rapid response and stroke teams following sudden bilateral cortical blindness enabled prompt brain imaging and diagnosis. Although MRI is more sensitive, stroke-series CT with perfusion allowed for rapid diagnosis in this critically ill patient [13]. Timely blood pressure control and tacrolimus cessation led to a favorable outcome without significant neurological sequelae.

## Conclusion

To our knowledge, this is the first reported case of PRES in a bilateral lung transplant recipient occurring in the setting of subtherapeutic tacrolimus levels, thereby challenging the typical association of PRES with elevated immunosuppressant concentrations. This case underscores the complex interplay between tacrolimus neurotoxicity, predisposing risk factors, blood–brain barrier integrity, and individual pharmacogenetic variability. Early recognition and prompt management of PRES are crucial to improving patient outcomes. Furthermore, this case broadens the clinical spectrum of PRES by identifying lung transplant recipients as a potential at-risk group and highlights the need for heightened clinical vigilance even when immunosuppressant levels are subtherapeutic.

## Data Availability

No datasets were generated or analyzed during the current study.

## Abbreviations

PRES: Posterior reversible encephalopathy syndrome
CT: Computed tomography
MR: Magnetic resonance
BD: Twice a day
ICU: Intensive care unit
NIHSS: National Institutes of Health Stroke Scale
FLAIR: Fluid-attenuated inversion recovery
MTT: Mean transit time
CBV: Cerebral blood volume
BBB: Blood–brain barrier
